# Case Report: Therapeutic biopsy guided targeted therapy improves visual acuity and prolongs survival in bilateral uveal metastases as the initial presentation of lung adenocarcinoma

**DOI:** 10.3389/fmed.2025.1643966

**Published:** 2025-10-10

**Authors:** Tongtong Ou, Yiliu Yang, Chengda Ren, Fang Lu

**Affiliations:** Department of Ophthalmology, West China Hospital, Sichuan University, Chengdu, Sichuan Province, China

**Keywords:** bilateral uveal metastasis, biopsy, case report, entrectinib, lung adenocarcinoma

## Abstract

**Background:**

Although uveal metastasis is common in lung cancer, reports of bilateral uveal metastasis as the initial symptom in lung adenocarcinoma patients undergoing intraocular biopsy are rare. Such cases typically portend poor outcomes, with a median survival of only 6.5 months. The current diagnostic gold standard involves intraocular biopsy (vitreous or choroidal), which carries significant risks, including tumor dissemination and retinal detachment. Crucially, these procedures are purely diagnostic, requiring additional interventions for local tumor control. This gap underscores the need for innovative strategies that integrate diagnosis, local therapy, and molecular profiling without compromising ocular function or survival outcomes.

**Case presentation:**

A 40-year-old male patient presented to our hospital with blurred vision and hemianopia in his right eye. Ophthalmic examination revealed bilateral uveal masses. Subsequent CT and PET/CT scans identified lung cancer with multiple metastases. Percutaneous biopsy of the lung confirmed the diagnosis of stage 4B lung adenocarcinoma. Transscleral biopsy was performed on the tumor in the left eye without affecting vision, and its immunohistochemical results were consistent with lung adenocarcinoma. Following transscleral tumor therapy and EGFR-TKI therapy, the patient achieved a best corrected visual acuity (BCVA) of 20/20 in both eyes with controlled tumor progression, surviving with stable disease for 3 years.

**Conclusion:**

This case underscores that bilateral uveal metastasis, even with ciliary body involvement, can achieve exceptional survival and visual recovery through targeted therapy. This case suggests ocular biopsy and early molecular testing may refine management for similar patients. The unexpected treatment response warrants further studies on tumor-ocular microenvironment interactions.

## 1 Introduction

Metastatic cancer is the most common type of intraocular malignancy (1). In lung adenocarcinoma, ocular metastases typically occur in advanced stages and often indicate poor prognosis, with a median survival of only 6.5 months (2). While the choroid (88%) is the most frequently affected site, metastasis to the ciliary body (2%) remains exceptionally rare and poses diagnostic challenges due to its atypical presentation (3).

Bilateral uveal metastases as the initial manifestation of lung cancer are scarcely reported, and their misdiagnosis risk is high, especially when visual symptoms are unilateral. Current management relies heavily on systemic imaging, yet confirmatory intraocular biopsies are rarely performed due to procedural risks. This gap underscores the need for innovative diagnostic approaches and tailored therapies to improve outcomes.

Here, we present a case of advanced lung adenocarcinoma initially manifesting as unilateral vision loss and bilateral uveal metastases including ciliary body involvement, diagnosed by transscleral biopsy and treated with targeted therapy that defies conventional prognostic expectations, resulting in 3-year survival with a BCVA of 20/20.

## 2 Case presentation

**Figure 1:**
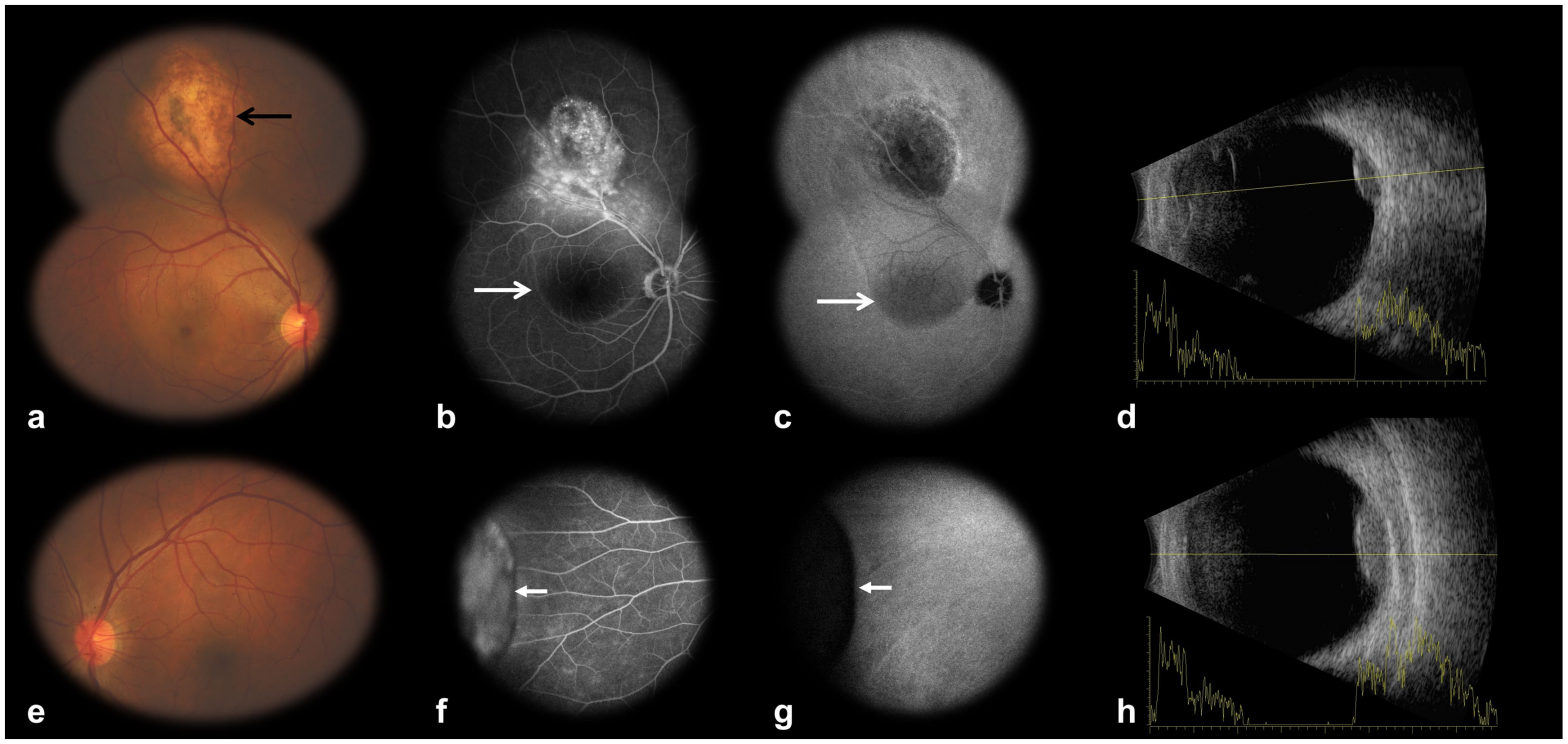
Multiple ophthalmic imaging of bilateral uveal masses. **(a)** Non-mydriatic fundus photography reveals a subretinal mass in the temporal-superior quadrant of the right eye (black arrow). **(b,c)** FA demonstrates hyperfluorescence of the right eye mass with late-phase leakage, while ICGA shows hypofluorescence. Note associated subretinal fluid in the macula (white long arrow). **(d)** B-scan ultrasonography depicts an oval, posteriorly attached mass in the right eye. **(e)** Fundus photography of the left eye (non-panoramic) failed to capture the ciliary body mass due to its extreme peripheral location. **(f,g)** FA and ICGA of the left eye nasal periphery (white short arrow) exhibit hyperfluorescence and hypofluorescence, respectively. **(h)** B-scan ultrasonography identifies a plateau-shaped mass adherent to the left ciliary body. FA, fluorescein angiography; ICGA, indocyanine green angiography.

A 40-year-old male patient presented with a complaint of blurred vision and diplopia in the right eye for 12 days. He denied symptoms such as cough, sputum production, or hemoptysis. He denied any previous ophthalmologic disorders or interventions. He had a history of smoking, and his father had been diagnosed with rectal cancer. Upon examination, the BCVA in the right eye was 20/50, while the left eye was 50/50. The left eye conjunctiva was congested and swollen. Fundus examination revealed subretinal masses in both eyes. The mass in the right eye was located in the temporal-superior choroid, with subretinal fluid in the posterior segment (Figure 1a). Another mass was found in the left eye, situated at the nasal periphery, at the junction of the choroid and ciliary body (Figure 1e). Fluorescein angiography (FA) revealed hyperfluorescent lesions with vascular leakage (Figures 1b,f), demonstrating localized dye extravasation that progressed from faint to moderate intensity. Notably, the right macula exhibited hypofluorescence due to blockage from subretinal fluid. In contrast, indocyanine green angiography (ICGA) showed consistent hypofluorescence (Figures 1c,g), indicating choroidal tumor localization behind the retinal pigment epithelium. These findings collectively confirmed uveal tract involvement with secondary retinal detachment. B-scan ultrasonography revealed an oval mass on the right posterior ocular wall and a plateau-shaped lesion adherent to the left ciliary body (Figures 1d,h). Optical coherence tomography (OCT) images were consistent with this assessment (not pictured). The patient received transpupillary thermotherapy (TTT) for the right eye to reduce the subretinal fluid in the macula and improve vision in the right eye.

**Figure 2:**
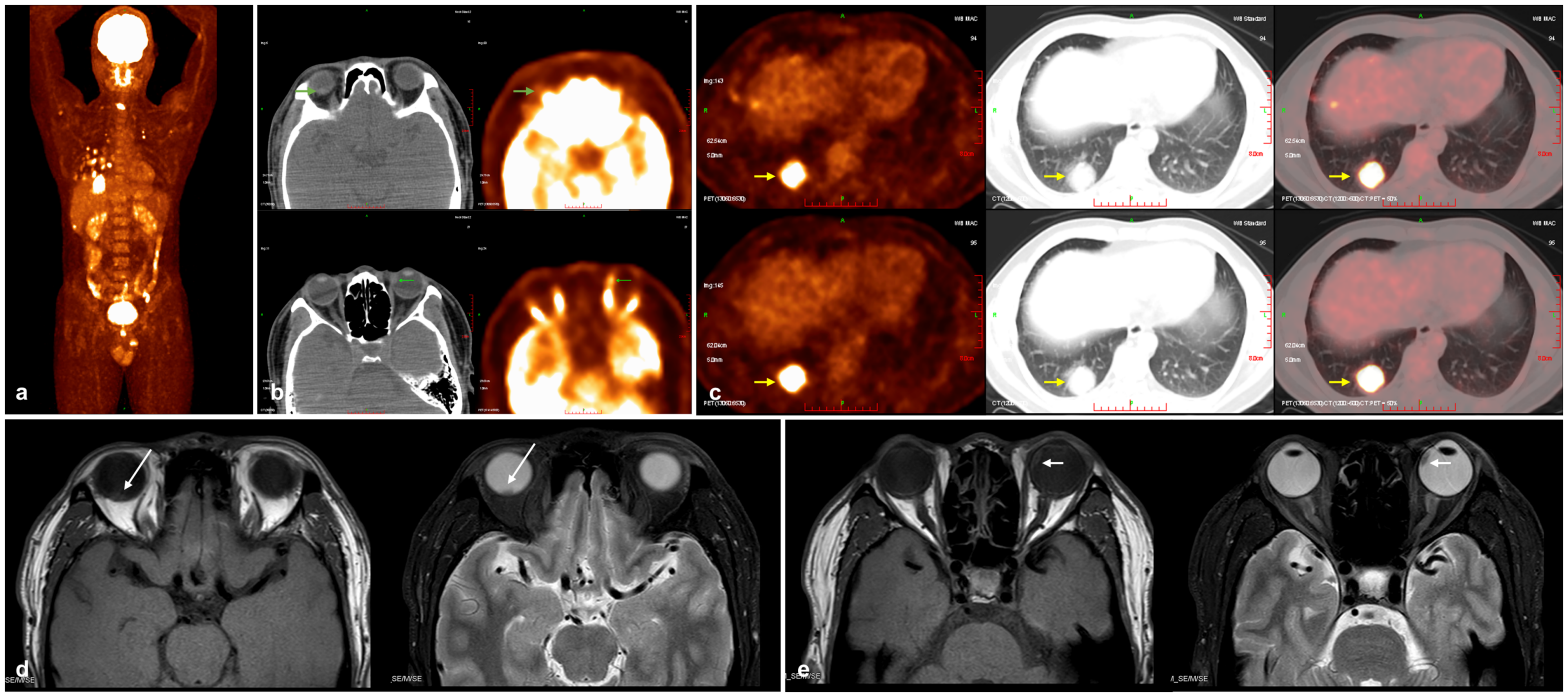
Whole-body FDG PET/CT and orbital MRI scans. **(a)** FDG PET/CT (maximum intensity projection) demonstrates hypermetabolic lesions in bronchial lymph nodes, skeletal system, and lungs. **(b)** FDG PET/CT shows increased FDG uptake in the masses (green arrow). **(c)** The largest pulmonary mass is located in the posterior basal segment of the right lower lobe (yellow arrow). **(d,e)** MRI scans reveal heterogeneous isointense signals in the lesions of both eyes on T1-weighted images, and heterogeneous hypointense signals on T2-weighted images (right eye indicated by a white long arrow, left eye indicated by a white short arrow). FDG PET/CT, fluorodeoxyglucose positron emission tomography/computed tomography; MRI, magnetic resonance imaging.

Suspecting uveal metastatic carcinoma, a full-body tumor screening was performed for the patient. Chest computed tomography (CT) revealed multiple nodules and masses of varying sizes in both lungs, with the largest measuring approximately 3.4 × 3.3 cm. Fluorodeoxyglucose positron emission tomography/computed tomography (FDG PET/CT) imaging demonstrated hypermetabolic lesions in the bronchial lymph nodes and bones, alongside the known pulmonary and ocular masses (Figure 2a). The maximum standardized uptake values (SUVmax) for the right and left eye masses were quantified as 2.19 (right eye), 6.69 (left eye), and 13.08 (dominant pulmonary mass) (Figures 2b,c). However, nuclear medicine specialists considered the possibility of primary bilateral choroidal melanoma with pulmonary and skeletal metastasis. The MRI scans demonstrated well-circumscribed intraocular masses with heterogeneous isointensity relative to vitreous on T1-weighted imaging and heterogeneous hypointensity on T2-weighted imaging, observed bilaterally (Figures 2d,e). Following confirmation of adenocarcinoma through percutaneous lung biopsy and a diagnosis of stage 4B lung adenocarcinoma (T4N3M1c), though respiratory specialists recommended excluding primary uveal tumors given the ocular SUVmax fell within the overlap range between metastases and primary melanoma.

**Figure 3:**
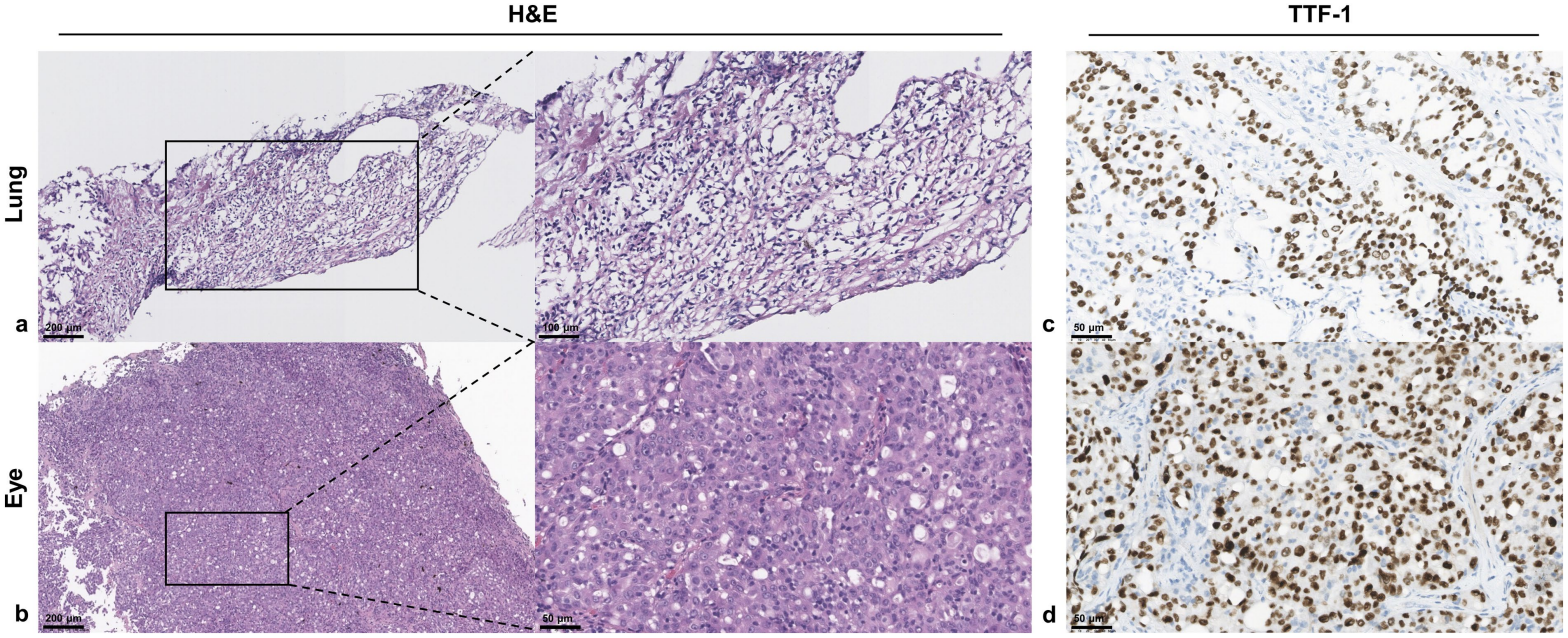
Histopathological and immunohistochemical characterization of biopsy specimens from pulmonary and left ocular masses. **(a)** H&E staining of the pulmonary biopsy reveals pleomorphic tumor cells with foamy cytoplasm and prominent nucleoli. Left: ×10 magnification; Right: ×20 magnification. **(b)** H&E staining of the ocular biopsy reveals malignant cells (black arrow) within a richly vascularized stroma (white arrowheads), indicative of tumor-induced angiogenesis. Left: ×10 magnification; Right: ×40 magnification. **(c,d)** Immunohistochemical confirmation of pulmonary origin. TTF-1 positivity in both **(c)** lung and **(d)** ocular metastases. Images acquired at ×40 magnification. H&E, hematoxylin and eosin; TTF-1, thyroid transcription factor-1.

To further determine the nature of the uveal tumor, a transscleral biopsy was performed on the tumor in the left eye. Due to its superficial position and limited size, the tumor was resected with full preservation of retinal integrity and visual acuity. Hematoxylin–eosin staining shows pleomorphic tumor cells with foamy cytoplasm in both the lung mass and left eye mass, with the eye mass additionally demonstrating scattered pigment deposition and delicate capillaries. Thyroid transcription factor-1 (TTF-1) immunohistochemical staining reveals nuclear positivity in tumor cells from both sites. The consistent pathological features and immunohistochemical results support the diagnosis of metastatic pulmonary adenocarcinoma (Figure 3).

Subsequently, the patient underwent next-generation sequencing (NGS) for targeted therapy-related gene mutations using the tissue from the percutaneous lung biopsy. The NGS results revealed an *EZR-ROS1* fusion with a mutation frequency of 42.99%, providing guidance for the patient’s drug selection. After confirming the diagnosis of metastatic lung cancer, the patient enrolled in a clinical study at our hospital’s respiratory department, receiving systemic treatment with the ROS1 kinase inhibitor entrectinib for lung cancer at a dose of 600 mg daily. After approximately 2 months, the patient’s overall tumor status was evaluated.

**Figure 4:**
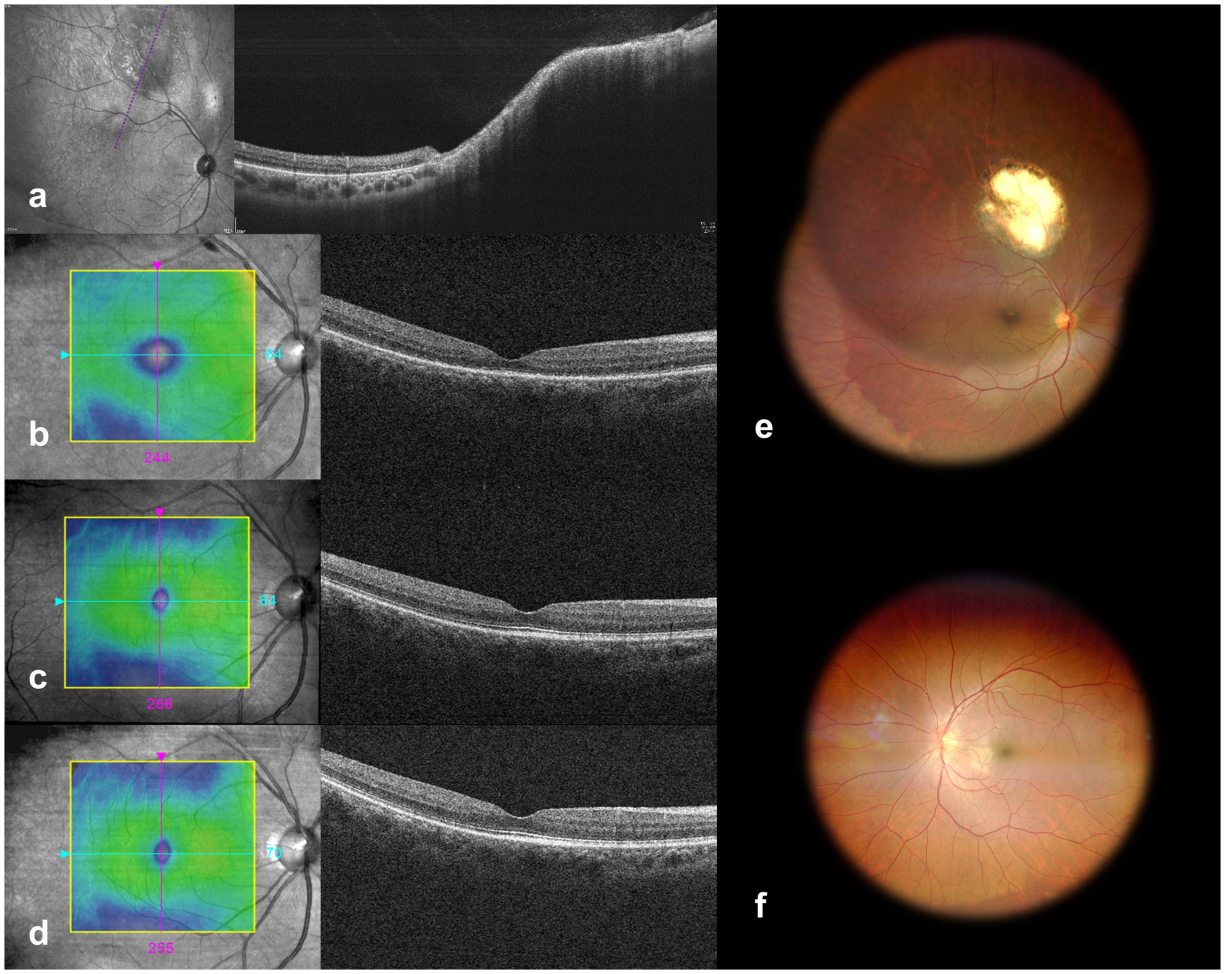
Post-therapeutic ophthalmic imaging of the right eye. **(a)** OCT of the right eye at 2 months shows persistent choroidal elevation (purple reference line) with retinal architectural disruption but resolved subretinal fluid. **(b–d)** Sequential macular OCT at 1, 19, and 28 months post-TTT demonstrates preserved foveal architecture with normal retinal thickness, confirming sustained therapeutic efficacy. **(e,f)** Final fundus photography at 28 months revealed **(e)** stable tumor dimensions without progression in the right eye and preserved macular architecture, while **(f)** the left eye showed no residual lesions or fundus abnormalities post-excisional biopsy. OCT, optical coherence tomography; TTT, transpupillary thermotherapy.

Two months after TTT treatment in the right eye, OCT revealed almost complete resolution of the subretinal fluid caused by tumor exudation, with choroidal elevation in the temporal-superior area of the mass, indicating that the mass was located within the choroid (Figure 4a). Prior to TTT, fundus photography of the right eye displayed significant areas of exudation. Follow-up after treatment revealed a reduction in the size of the exudative area (Figure 4e), indicating that TTT treatment was effective in controlling the exudation. Sequential observations showed that OCT of the macula revealed that no subretinal fluid was present, and the retinal structure remained intact (Figures 4b–d), supporting the assessment of a healthy macula and having a positive impact on postoperative visual recovery. After undergoing transscleral resection of the left eye mass, fundus photography examination showed complete resolution of the original lesion, with a clear surgical field and no obvious signs of recurrence, suggesting a successful surgery and effective removal of the lesion (Figure 4f).

To date, the patient has adhered to the research protocol, continuing treatment with entrectinib for about 3 years and regularly returning to the hospital for follow-up visits. Systemic evaluations were conducted approximately every 3 months in accordance with the lung cancer center’s protocol. These assessments included CT of the pelvis, chest, and abdomen, as well as MRI of the head, to monitor disease status. Ophthalmic follow-up examinations were performed at intervals of 6–12 months, considering the patient’s stable visual function and well-controlled systemic disease. These evaluations included measurements of visual acuity, intraocular pressure, and fundus examination. The patient’s overall tumor status remains well-controlled, with the most recent tumor assessment showing partial remission. During a recent ophthalmology follow-up, a yellow-white oval lesion was observed in the temporal-superior quadrant of the right eye, while no lesions were detected in the left eye. Best corrected visual acuity in both eyes was 20/20. The patient is satisfied with the improvement in their vision. The patient reported no subjective discomfort or side effects throughout the treatment period, and no adverse or unanticipated events were observed by the clinical team. We will continue to monitor the patient.

## 3 Discussion

Among the uveal structures, the choroid is the most frequently affected due to its rich blood supply (4). In a study of 194 cases of uveal metastasis from lung cancer, approximately 88% of metastases were located in the choroid (5). The most common type of lung cancer to cause choroidal metastasis is adenocarcinoma, followed by squamous cell carcinoma. The primary cancers most commonly leading to choroidal metastasis include breast cancer and lung cancer (6). Bilateral, multifocal metastasis is most often secondary to breast cancer, while unilateral, solitary metastasis is more commonly seen in lung cancer. However, the eyes are not routinely screened for metastatic tumors, and intraocular metastases that do not involve the visual functional areas may remain undiagnosed, and thus only 35% of ocular metastases are detected prior to the diagnosis of lung cancer (7).

For this patient, although he had severe extraocular metastases involving the lungs, bones, and brain, the ocular symptoms were the initial manifestation of lung adenocarcinoma. Compared to other metastatic sites, ocular symptoms may aid in the early diagnosis of lung cancer. Pathological examination of the left eye mass confirmed that it shared the same histological origin as the lung cancer (TTF-1 positive, lung adenocarcinoma). In this case, the diagnosis of choroidal metastatic carcinoma preceded the diagnosis of systemic cancer, which is most commonly seen in lung cancer (8). In a study by Shah et al., 44% of patients diagnosed with uveal metastasis from lung cancer had no prior history of lung cancer, and misdiagnosis was not uncommon, with 14% of patients initially referred with a diagnosis of uveal melanoma (5).

In this case, the masses in both eyes were located in different areas of the uvea. The tumor in the left eye was located in the ciliary body, causing pressure symptoms without significant visual impairment. The patient had no symptoms suggestive of a primary lung cancer or history of malignancy, presenting with blurred vision and hemianopia in one eye, which could have been misdiagnosed as uveal melanoma. However, primary uveal melanoma typically presents as a unilateral lesion, with bilateral primary uveal melanoma being exceedingly rare (9). Similarly, melanoma metastasizing to the contralateral eye is uncommon and usually manifests as a diagnosis in one eye followed by subsequent involvement in the opposite eye (10). Therefore, we are inclined not to attribute the bilateral involvement to primary uveal melanoma. Additionally, uveal melanoma can undergo hematogenous spread, with the liver affected in 90% of metastatic cases (11). However, the patient’s liver was not involved.

Metastatic uveal tumors and primary tumors may present similarly in clinical practice, with most patients presenting with blurred vision, typically associated with macular or retinal involvement, or serous retinal detachment in the fovea. Compared to choroidal melanoma, metastatic tumors are less likely to present with ocular flashes, floaters, or visual field defects (8).

In most cases, ophthalmologists can differentiate metastatic uveal tumors from primary tumors (such as melanoma) through fundoscopy, angiography, and OCT.

Metastatic tumors typically appear on fundoscopy as whitish or pale yellow masses with subretinal fluid, except in the case of metastatic melanoma (6). Differences in growth patterns and tissue architecture further contribute to their distinct appearance on examination. Choroidal metastatic carcinoma develops rapidly, diffusely infiltrating normal choroidal tissue, with solid glandular structures that produce high echogenicity on imaging. In contrast, melanomas grow more slowly, forming mushroom-shaped choroidal masses that typically breach Bruch’s membrane, with dense cellular clusters that exhibit low to medium homogeneous echogenicity (8).

FA features of metastatic lesions, including early hypofluorescence with late-phase staining, are non-specific, making it difficult to differentiate uveal metastasis from primary tumors (12). However, the relatively avascular nature of most metastases may facilitate their differentiation from other choroidal tumors such as choroidal hemangioma (13). ICGA in metastatic carcinoma typically shows choroidal fluorescence blockage and patchy surface staining (4). On OCT, surface irregularities are a characteristic feature of choroidal metastatic carcinoma, whereas melanoma presents as a dome-shaped tumor with fluffy photoreceptor layers, choroidal nevi show chronic overlying retinal atrophy, and hemangiomas display enlarged choroidal vascular lumens. Choroidal osteoma, in contrast, appears as a thin-layered bony structure (14). Additionally, OCT can assess the optical density of subretinal fluid, which is significantly lower in metastatic carcinoma compared to melanoma (15). Mathis et al. proposed that subretinal fluid in the early stages of disease has lower protein concentrations and optical density than in later stages, as metastatic carcinoma is often located at the posterior pole and presents early, whereas choroidal melanoma tends to be located peripherally and remains asymptomatic for a long time. Consequently, metastatic carcinoma is often detected at an earlier stage of the disease (8).

When it is difficult to determine the nature of a uveal tumor or primary tumor, intraocular tumor biopsy may be required, though it carries risks such as tumor dissemination and retinal detachment (16). While intraocular biopsies raise concerns about seeding, this case demonstrates that transscleral excision of appropriately selected tumors (small, anteriorly located) can mitigate such risks while achieving diagnostic certainty. Given the tumor’s small size and favorable location in the ciliary body, complete transscleral excision served both therapeutic and diagnostic purposes, enabling tumor removal while providing tissue for histopathological confirmation. Intraocular biopsy not only confirms the tissue origin but also identifies specific molecular alterations to guide targeted therapies. An emerging non-invasive diagnostic method is liquid biopsy, such as blood for lung cancer or vitreous for intraocular tumors (17, 18).

For lung cancer patients with intraocular metastasis, systemic therapy can yield favorable outcomes. These results are consistent with a recent systematic review and pooled analysis (19), which further confirms the survival benefits of targeted therapy in this patient population. Entrectinib is an orally active, small-molecule selective multi-target inhibitor that targets tropomyosin receptor kinase (*TRK*), the proto-oncogene tyrosine-protein kinase ROS1 (*ROS1*), and anaplastic lymphoma kinase (*ALK*) (20). Additionally, entrectinib has unique central nervous system penetrability and can cross the blood–brain barrier, making it the only TRK inhibitor clinically proven to be effective against both primary and metastatic brain diseases (21). In this case, the patient’s most recent tumor assessment showed partial remission, and he has survived for over 30 months since the diagnosis of metastatic lung cancer, which is superior to the median duration of response (DoR) of 13.0 months and the median progression-free survival (PFS) of 12.9 months reported in prior study of entrectinib for *ROS1*-positive advanced NSCLC (22). We intend to perform deoxyribonucleic acid (DNA) sequencing on the tumor from the left eye, which was removed via scleral excision. If *ROS1*-positivity is detected, it will further confirm the therapeutic mechanism of entrectinib in suppressing ocular tumors.

In addition to the systemic targeted therapy, the management of ocular metastases requires careful consideration of adjuvant strategies to preserve optic nerve function, particularly in cases threatening compression or ischemia. The immediate administration of pulsed dose corticosteroids could be employed to reduce perineural edema and inflammatory damage, potentially providing a critical window for neural recovery prior to the onset of irreversible ischemia (23). For more definitive local control, radioactive patch therapy offers a means to deliver targeted radiation directly to the tumor bed, achieving effective decompression while minimizing exposure to surrounding healthy structures such as the optic nerve (24). Furthermore, emerging biologic agents targeting angiogenesis and vascular permeability, such as faricimab against VEGF and angiopoietin pathways, have shown prolonged control in other ocular pathologies (25). Although our patient had no optic nerve compromise, thanks to systemic therapy, these adjuvant options remain vital in multidisciplinary ocular metastasis management. Therefore, these therapeutic approaches represent a complete therapeutic avenue that requires comprehensive consideration in the context of oncologic emergencies affecting the visual pathway.

This study has several limitations inherent to its design and setting. Firstly, as a single-case report, its findings cannot be generalized to a broader population without further validation in larger cohorts. Secondly, the other limitation is the lack of certain specialized on-site monitoring technologies. Although we monitored response through standard ophthalmologic examination and conventional imaging, we did not have access to more nuanced tools. For example, serial liquid biopsy analysis for circulating tumor DNA (ctDNA) could have provided real-time, molecular-level tracking of treatment response and emerging resistance (26), thereby limiting our analysis to clinical and radiological findings. Nonetheless, this case highlights how a timely, biomarker-guided biopsy directly informed therapy that preserved both vision and life. Future efforts should prioritize validating this approach in similar scenarios.

A review of recent clinical cases shows that this case is of pioneering significance. It is the first to demonstrate the successful application of biopsy and targeted therapy in a advanced lung cancer patient with bilateral uveal metastases as the initial manifestation. Not only did this result in a partial remission of the lung cancer, but the patient’s BCVA in both eyes reached 20/20. This case highlights the critical role of ophthalmologists in recognizing ocular metastasis from non-ocular primary tumors, and underscores the importance of multidisciplinary collaboration in managing such complex cases. It demonstrates the clinical feasibility of achieving tumor control while preserving vision and opens new pathways for the treatment of similar patients.

## Data Availability

The original contributions presented in the study are included in the article/supplementary material, further inquiries can be directed to the corresponding author.

## Glossary

ALK: anaplastic lymphoma kinase
ATP: adenosine triphosphate
BCVA: best corrected visual acuity
CT: computed tomography
ctDNA: circulating tumor deoxyribonucleic acid
DNA: deoxyribonucleic acid
DoR: duration of response
EGFR-TKI: epidermal growth factor receptor tyrosine kinase inhibitor
FA: fluorescein angiography
FDG PET/CT: fluorodeoxyglucose positron emission tomography/computed tomography
ICGA: indocyanine green angiography
MRI: magnetic resonance imaging
NGS: next-generation sequencing
NSCLC: non-small cell lung cancer
OCT: optical coherence tomography
ORR: objective response rate
OS: overall survival
PFS: progression-free survival
ROS1: ROS proto-oncogene 1, receptor tyrosine kinase
SUVmax: maximum standardized uptake values
TRK: tropomyosin receptor kinase
TTF-1: thyroid transcription factor-1
TTT: transpupillary thermotherapy
VEGF: vascular endothelial growth factor
