# Supplementary material for: Case Report: Therapeutic biopsy guided targeted therapy improves visual acuity and prolongs survival in bilateral uveal metastases as the initial presentation of lung adenocarcinoma
Source: Front Med (Lausanne). 2025 Oct 10;12:1643966. doi: 10.3389/fmed.2025.1643966 (PMC12549678; doi:10.3389/fmed.2025.1643966)
Supplement: Supplementary file 1 [file Data_Sheet_1.pdf]

CARE case report guidelines

CARE Checklist of information to include when writing a case report

(cc) BY-NC-ND

Topic	Item	Checklist item description	Reported on Line
Title	1	The diagnosis or intervention of primary focus followed by the words "case report"	√
Key Words	2	2 to 5 key words that identify diagnoses or interventions in this case report, including "case report"	102
Abstract (no references)	3a	Introduction: What is unique about this case and what does it add to the scientific literature?	74–83
	3b	Main symptoms and/or important clinical findings	84–88
	3c	The main diagnoses, therapeutic interventions, and outcomes	88–93
	3d	Conclusion—What is the main "take-away" lesson(s) from this case?	94–99
Introduction	4	One or two paragraphs summarizing why this case is unique (**may include references**)	111–129
Patient Information	5a	De-identified patient specific information.	134
	5b	Primary concerns and symptoms of the patient.	134–135
	5c	Medical, family, and psycho-social history including relevant genetic information	135–138
	5d	Relevant past interventions with outcomes	137–138
Clinical Findings	6	Describe significant physical examination (PE) and important clinical findings.	167–188
Timeline	7	Historical and current information from this episode of care organized as a timeline	Supplementary material
Diagnostic Assessment	8a	Diagnostic testing (such as PE, laboratory testing, imaging, surveys).	259–261, 265–266
	8b	Diagnostic challenges (such as access to testing, financial, or cultural)	261–264
	8c	Diagnosis (including other diagnoses considered)	273–275
	8d	Prognosis (such as staging in oncology) where applicable	111–114
Therapeutic Intervention	9a	Types of therapeutic intervention (such as pharmacologic, surgical, preventive, self-care)	185–188, 265–268, 281–311
	9b	Administration of therapeutic intervention (such as dosage, strength, duration)	281–311
	9c	Changes in therapeutic intervention (with rationale)	311–465
Follow-up and Outcomes	10a	Clinician and patient-assessed outcomes (if available)	265–276
	10b	Important follow-up diagnostic and other test results	311–465
	10c	Intervention adherence and tolerability (How was this assessed?)	322–340
	10d	Adverse and unanticipated events	461–465
Discussion	11a	A scientific discussion of the strengths AND limitations associated with this case report	470–496
	11b	Discussion of the relevant medical literature **with references**.	470–624
	11c	The scientific rationale for any conclusions (including assessment of possible causes)	470–624
	11d	The primary "take-away" lessons of this case report (without references) in a one paragraph conclusion	625–636
Patient Perspective	12	The patient should share their perspective in one to two paragraphs on the treatment(s) they received	461–462
Informed Consent	13	Did the patient give informed consent? Please provide if requested	Yes ☑ No ☐
