# Supplementary material for: Case Report: Therapeutic biopsy guided targeted therapy improves visual acuity and prolongs survival in bilateral uveal metastases as the initial presentation of lung adenocarcinoma
Source: Front Med (Lausanne). 2025 Oct 10;12:1643966. doi: 10.3389/fmed.2025.1643966 (PMC12549678; doi:10.3389/fmed.2025.1643966)
Supplement: Supplementary file 2 [file Table_1.docx]

## Table showing the timeline of cares

| **Date** | **Event** | **Outcome** |
| --- | --- | --- |
| Jan. 2022 | Right eye blurred vision (12 days) | Initial presentation; diagnostic workup initiated. |
|  | Imaging and biopsy | Bilateral choroidal masses (suspected malignancy).  Pulmonary nodules/masses (CT).  PET/CT: Metastases (lungs, bones, lymph nodes).  Biopsy confirmed: Right lung adenocarcinoma (TTF-1 positive). |
| Feb. 2022 | Molecular testing | EZR-ROS1 fusion detected. |
|  | Ophthalmic interventions | Right eye: TTT therapy.  Left eye: Tumor resection biopsy (TTF-1 positive). |
| Apr. 2022 | Initiated entrectinib trial | Dose: 600 mg/day. |
| 2022–2025 | Regular follow-up | Imaging (CT/MRI): Sustained partial response (PR), no progression.  Ophthalmology: Stable vision (20/20), OCT structurally stable. |
